# Predicting the risk of lean non-alcoholic fatty liver disease based on interpretable machine models in a Chinese T2DM population

**DOI:** 10.3389/fendo.2025.1626203

**Published:** 2025-07-11

**Authors:** Shixue Bao, Qiankai Jin, Tieqiao Wang, Yushan Mao, Guoqing Huang

**Affiliations:** ^1^ Department of Endocrinology, The First Affiliated Hospital of Ningbo University, Ningbo, Zhejiang, China; ^2^ Department of Endocrinology, Beilun People's Hospital, Ningbo, Zhejiang, China

**Keywords:** lean non-alcoholic fatty liver disease, type 2 diabetes mellitus, interpretable machine learning, prediction model, predict risk

## Abstract

**Background:**

Non-alcoholic fatty liver disease (NAFLD) is the most common chronic liver disease, seriously threatening the public health. Although the proportion of patients with lean NAFLD is lower than that of patients with obese NALFD, it should not be overlooked. This study aimed to construct interpretable machine learning models for predicting lean NAFLD risk in type 2 diabetes mellitus (T2DM) patients.

**Methods:**

This study enrolled 1,553 T2DM individuals who received health care at the First Affiliated Hospital of Ningbo University, Ningbo, China, from November 2019 to November 2024. Feature screening was performed using the Boruta algorithm and the Least Absolute Shrinkage and Selection Operator (LASSO). Linear discriminant analysis (LDA), logistic regression (LR), Naive Bayes (NB), random forest (RF), support vector machine (SVM), and extreme gradient boosting (XGboost) were used in constructing risk prediction models for lean NAFLD in T2DM patients. The area under the receiver operating characteristic curve (AUC) was used to assess the predictive capacity of the model. Additionally, we employed SHapley Additive exPlanations (SHAP) analysis to unveil the specific contributions of individual features in the machine learning model to the prediction results.

**Results:**

The prevalence of lean NAFLD in the study population was 20.3%. Eight variables, including age, body mass index (BMI), and alanine aminotransferase (ALT), were identified as independent risk factors for lean NAFLD. Ten predictive factors, including BMI, ALT, and aspartate aminotransferase (AST), were screened for the construction of risk prediction models. The random forest model demonstrated superior performance compared to alternative machine learning (ML) algorithms, achieving an AUC of 0.739 (95% confidence interval [CI]: 0.676–0.802) in the training set, and it also exhibited the best predictive value in the internal validation set with an AUC of 0.789 (95% CI: 0.722–0.856). In addition, the SHAP method identified TG, ALT, GGT, BMI, and UA as the top five variables influencing the predictions of the RF model.

**Conclusion:**

The construction of lean NAFLD risk models based on the Chinese T2DM population, particularly the RF model, facilitates its early prevention and intervention, thereby reducing the risks of intrahepatic and extrahepatic adverse outcomes.

## Introduction

In recent years, type 2 diabetes mellitus (T2DM) and Non-alcoholic fatty liver disease (NAFLD) have been the two most challenging public health issues worldwide. T2DM is a metabolic disease characterized by chronic elevated blood glucose caused by many factors, such as heredity and environment. Approximately 537 million (10.5%) adults (aged 20–79) in the world have T2DM according to the latest research results of the International Diabetes Federation (IDF) (1). NAFLD refers to a clinicopathological syndrome primarily characterized by excessive intrahepatic fat deposition, excluding alcohol consumption and other well-defined liver-damaging factors (2). It is estimated that the global prevalence of NAFLD has increased from 25% in 2016 to over 30% at present and continues to rise (3, 4). It is well-known that there is a close relationship between T2DM and NAFLD. They not only share common risk factors but also frequently serve as comorbidities or target organ damages for each other. T2DM and NAFLD are known to frequently coexist and act synergistically to increase the risk of adverse clinical outcomes (5). A meta-analysis revealed that the prevalence rates of NAFLD in T2DM were 65.04% (95% CI:61.79%–68.15%), and the prevalence of NAFLD in the Chinese population with T2DM is 52.56% (6). Another study result showed that compared with T2DM patients without NAFLD, the risks of cardiovascular disease (CVD), chronic kidney disease (CKD) and proliferative retinopathy in T2DM patients with NAFLD are 1.96, 1.87 and 1.75 times higher respectively (7). Furthermore, it has been demonstrated that patients with NAFLD exhibit a more than twofold increased risk of developing T2DM compared to the general population (5).

Although NAFLD is commonly associated with obesity, around 10%–20% of NAFLD cases occur in non-obese or non-overweight individuals, a condition often described as lean NAFLD (8). A Meta-analysis encompassing 33 observational studies reported that the prevalence of lean NAFLD was the highest among Asian individuals (9). Compared to obese NAFLD, lean NAFLD has milder metabolic abnormalities but a higher incidence of advanced liver disease and all-cause mortality (10, 11). Due to the absence of obesity phenotypes, lean NAFLD is prone to being overlooked in clinical diagnosis. Currently, there are no formal recommendations for the treatment of lean NAFLD (12), early lifestyle intervention remains the cornerstone of the management of lean NAFLD (13). Certain relevant guidelines recommend that it is necessary to conduct screening for NAFLD in T2DM patients (14, 15). Therefore, it is particularly crucial to conduct early identification and effective management of lean NAFLD in a Chinese T2DM population.

The approach for lean NAFLD diagnosis is identical to any NAFLD patient. Excluding excessive alcohol consumption and other causes of hepatic steatosis and damage, detection is carried out through ultrasound, computed tomography (CT), or magnetic resonance (MR), and pathological diagnosis is performed using liver biopsy when necessary. Serum indices (NFS score and FIB-4 score) and imaging techniques (transient elastography and magnetic resonance elastography) can serve as alternative approaches to liver biopsy for fibrosis staging and patient follow-up (16–18). However, the above-mentioned diagnostic methods inevitably lead to the waste of medical resources and an increase in time costs. Therefore, it is of great significance to establish an assessment tool that can screen out high-risk individuals with lean NAFLD at an early stage.

With the development of technology, the utilization of artificial intelligence (AI) and machine learning (ML) technology in the healthcare sector has experienced significant growth in recent years (19). The analysis of extensive clinical data through ML algorithms can assist clinicians in identifying potential disease progression patterns and facilitating personalized treatment strategies (20). Currently, a substantial body of research has explored the utilization of various ML techniques for the prediction and diagnosis of diseases, such as T2DM, breast cancer, and heart disease (21–23). Pei-Yuan Su et al. developed ML models for the prediction of fatty liver disease in lean individuals. The results indicated that the ML model comprising a two-class neural network using 10 features had the highest area under the receiver operating characteristic curve (AUC) value (0.885) among all other algorithms (24). However, there are few studies on the risk prediction models for lean NAFLD in T2DM patients. The purpose of this study was to establish lean NAFLD risk prediction models based on interpretable machine learning algorithms, which would facilitate the early identification of lean NAFLD and guide appropriate preventive and intervention measures.

## Materials and methods

### Study population

**Figure 1:**
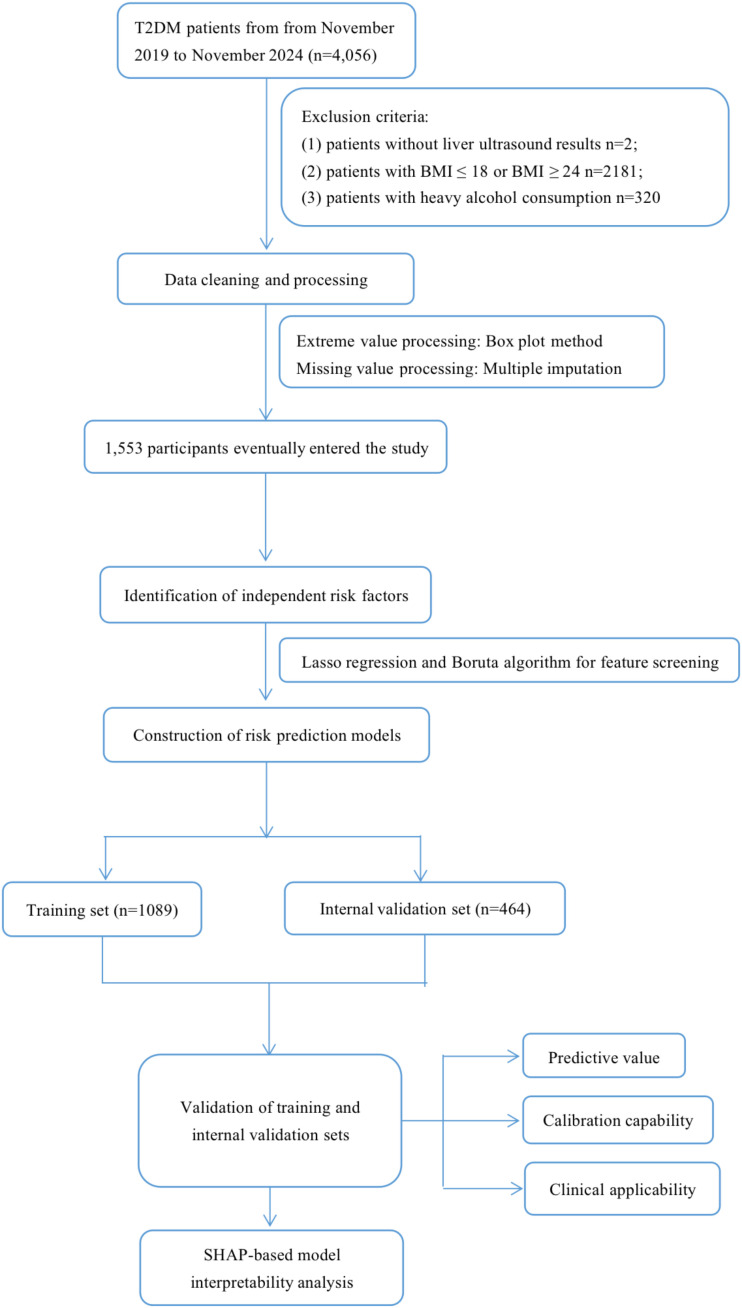
Flow diagram of the study.

This study enrolled 4,056 T2DM individuals who received health care through routine physical examinations, outpatient visits, and inpatient admissions at the First Affiliated Hospital of Ningbo University, Ningbo, China, from November 2019 to November 2024. Demographic data, relevant complications, biochemical parameters, etc., were obtained through questionnaire surveys and laboratory examinations. Ultimately, 1,553 participants were included in the study. The specific exclusion criteria were as follows: (1) patients without liver ultrasound results; (2) patients with body mass index (BMI) ≤ 18 or BMI ≥ 24; (3) patients with heavy alcohol consumption (exceeding 140 grams per week for men and 70 grams per week for women); (4) diagnosis of liver disease, such as viral hepatitis, and autoimmune hepatitis. We used the multiple imputation method (linear regression, polynomial logistic regression, and five iterations to create an interpolation model) to process missing values, in order to reduce bias toward missing data (**Supplementary Figure 1**) (25). The study’s flow diagram is depicted in **Figure 1**.

### Definition

The diagnostic criteria for DM were fasting blood glucose (FBG) levels of ≥ 7.0 mmol/L, 2-hour blood glucose levels of ≥ 11.1 mmol/L, or a glycated hemoglobin level of ≥ 6.5% (26).

### Clinical baseline data

Clinical baseline data encompassed the Participants’ general characteristics (gender, age, BMI, systolic blood pressure [SBP], diastolic blood pressure [DBP], and heart rate [HR]), lifestyle habits(history of smoking), blood cell counts (white blood cell count [WBC], red blood cell count [RBC], mean red blood cell volume [MCV], lymphocyte count [LYMPH], monocyte count[MONO], neutrophil count [NEUT], eosinophil count [EOS], basophil count [BASO], hemoglobin [HB], platelet count [PLT], mean platelet volume [MPV], and platelet distribution width [PDW]), biochemical indicators (alanine aminotransferase [ALT], aspartate aminotransferase [AST], gamma-glutamyl transpeptidase [GGT], total bilirubin [TBIL], direct bilirubin [DBIL], indirect bilirubin [IBIL], albumin [ALB], globulin [GLO], total protein [TP], creatinine [CREA], uric acid [UA], total cholesterol [TC], triglycerides [TG], high-density lipoprotein cholesterol [HDL-C], low-density lipoprotein cholesterol [LDL-C], and glucose [GLU]), and other laboratory value(glycosylated hemoglobin [HBA1C]).

### Statistical analysis

The preliminary analysis of the dataset involves the application of descriptive statistics. The Kolmogorov-Smirnov (K-S) test was used to test the normality of the continuous variables. Continuous variables with a normal distribution were expressed as means (standard deviations, SD), continuous variables with a skewed distribution were expressed as medians (interquartile ranges, IQR), and categorical variables were expressed as percentages (percentage, %). Independent-samples T test (continuous variables with a normal distribution), Mann-Whitney U test (continuous variables with a skewed distribution), and chi-square test (categorical variables) were used to evaluate the differences between groups, and the standardized mean difference (SMD) was used to evaluate balance between groups (27).

We used multivariate logistic regression to identify independent risk factors for lean NAFLD. The Boruta algorithm and the Least Absolute Shrinkage and Selection Operator (LASSO) were utilized to screen for characteristic variables. To construct a prediction model, the entire dataset was partitioned into a training set and an internal validation set at a ratio of 6:4. Subsequently, six ML algorithms including linear discriminant analysis (LDA), logistic regression (LR), Naive Bayes (NB), random forest (RF), support vector machine (SVM), and extreme gradient boosting (XGboost), were employed to train the model. During the model training process, a 10-fold cross-validation method was utilized to optimize the model parameters and prevent the occurrence of overfitting. In addition, we employed the AUC to evaluate the predictive ability of the model. Calibration curves and the Brier score were utilized to assess the calibration ability, while decision curve analysis (DCA) was applied to evaluate the clinical applicability. Additionally, the Shapley Additive exPlanations (SHAP) was used to interpret the best predictive model.

Statistical analyses were conducted using the R language (version 4.2.3, http://www.R-project.org/) and Python (version 3.9.0, https://www.python.org/). All data were analyzed using two-sided tests, and statistical significance was defined as *P* < 0.05.

## Results

### Baseline characteristics of participants

A total of 1,553 participants were recruited in this study, including 1,237 T2DM patients without lean NAFLD and T2DM 316 patients with complicated by lean NAFLD. The median age of the patients was 59.00 years (IQR:50.00–67.00), among whom 713 cases (45.9%) were male and 840 cases (54.1%) were female. When comparing the baseline characteristics between the two groups of patients, statistically significant differences were observed in terms of gender, BMI, DBP, routine blood tests, liver and kidney functions, blood lipid levels, and blood glucose levels (*p* < 0.05). As shown in **Table 1**.

**Table 1:** Univariate analysis of lean NAFLD.

	Overall	Normal	Lean NAFLD	*P*-value	SMD
N	1553	1237	316		
Gender (Male), %	713 (45.9)	569 (46.0)	144 (45.6)	0.942	0.009
Age, years	59.00 (50.00, 67.00)	59.00 (51.00, 67.00)	56.00 (45.00, 64.00)	<0.001	0.326
BMI, kg/m^2^	22.03 (20.76, 23.05)	21.80 (20.55, 22.89)	22.60 (21.64, 23.44)	<0.001	0.513
SBP, mmHg	130.00 (118.00, 144.00)	130.00 (118.00, 145.00)	130.00 (118.00, 142.00)	0.936	0.005
DBP, mmHg	77.00 (70.00, 85.00)	77.00 (70.00, 84.00)	79.00 (72.00, 87.00)	<0.001	0.22
HR, n	81.00 (75.00, 92.00)	81.00 (74.00, 91.00)	82.50 (76.00, 93.00)	0.113	0.121
Smoking (Yes), %	271 (17.5)	216 (17.5)	55 (17.4)	1	0.001
WBC, ×10^9^/L	6.53 (5.35, 7.90)	6.46 (5.20, 7.80)	6.97 (5.80, 8.30)	<0.001	0.133
RBC, ×10^12^/L	4.47 (4.11, 4.87)	4.40 (4.09, 4.80)	4.65 (4.29, 5.10)	<0.001	0.389
MCV, fL	90.60 (88.00, 93.40)	91.00 (88.00, 93.80)	90.00 (87.50, 92.60)	0.002	0.113
LYMPH, ×10^9^/L	1.70 (1.30, 2.20)	1.70 (1.30, 2.20)	1.90 (1.50, 2.32)	<0.001	0.059
MONO, ×10^9^/L	0.40 (0.31, 0.53)	0.40 (0.30, 0.51)	0.44 (0.37, 0.60)	0.031	0.082
NEUT, ×10^9^/L	4.00 (3.20, 5.20)	4.00 (3.10, 5.10)	4.20 (3.30, 5.50)	0.029	0.126
ESO, ×10^9^/L	0.08 (0.04, 0.14)	0.08 (0.04, 0.14)	0.09 (0.04, 0.13)	0.462	0.026
BASO, ×10^9^/L	0.03 (0.02, 0.04)	0.03 (0.02, 0.04)	0.03 (0.02, 0.04)	0.106	0.027
HB, g/L	130.00 (113.00, 143.00)	129.00 (112.00, 141.00)	135.00 (117.00, 149.25)	<0.001	0.078
PLT, ×10^9^/L	213.00 (177.00, 252.00)	211.00 (175.00, 251.00)	220.00 (188.00, 259.00)	0.006	0.116
MPV, fL	10.30 (9.60, 11.00)	10.30 (9.60, 11.10)	10.30 (9.70, 10.90)	0.895	0.021
PDW, %	12.10 (10.80, 13.90)	12.10 (10.70, 13.90)	12.00 (11.00, 14.00)	0.416	0.056
ALT, IU/L	19.00 (13.00, 28.00)	17.00 (13.00, 25.00)	25.50 (18.00, 35.00)	<0.001	0.312
AST, IU/L	19.00 (16.00, 25.00)	19.00 (15.00, 24.00)	22.00 (17.00, 28.00)	<0.001	0.099
GGT, U/L	21.00 (15.00, 34.00)	20.00 (14.00, 30.00)	31.00 (20.00, 46.25)	<0.001	0.165
TBIL, μmol/L	10.60 (8.00, 14.10)	10.40 (7.80, 13.70)	11.90 (8.80, 15.30)	<0.001	0.195
DBIL, μmol/L	3.00 (2.30, 4.00)	3.00 (2.20, 3.99)	3.10 (2.40, 4.12)	0.07	0.016
IBIL, μmol/L	7.50 (5.40, 10.20)	7.20 (5.20, 9.80)	8.40 (6.10, 11.40)	<0.001	0.292
ALB, g/L	41.30 (37.80, 44.70)	40.80 (37.50, 44.30)	43.10 (39.48, 45.73)	<0.001	0.359
GLO, g/L	28.10 (25.40, 31.50)	27.80 (25.30, 31.30)	28.90 (25.60, 31.72)	0.05	0.115
TP, g/L	69.90 (65.00, 74.50)	69.40 (64.60, 73.90)	71.65 (67.38, 75.90)	<0.001	0.349
CREA, μmol/L	60.00 (50.00, 74.00)	61.00 (50.60, 74.00)	59.00 (49.68, 75.00)	0.698	0.046
UA, μmol/L	295.00 (240.60, 357.00)	288.00 (237.00, 346.00)	325.65 (260.50, 398.08)	<0.001	0.384
TC, mmol/L	4.76 (4.01, 5.66)	4.69 (3.99, 5.57)	5.02 (4.12, 5.95)	<0.001	0.218
TG, mmol/L	1.30 (0.91, 1.91)	1.21 (0.85, 1.77)	1.69 (1.22, 2.67)	<0.001	0.423
HDL-C, mmol/L	1.16 (0.99, 1.39)	1.18 (1.01, 1.42)	1.12 (0.95, 1.33)	0.002	0.202
LDL-C, mmol/L	3.05 (2.42, 3.66)	3.00 (2.41, 3.60)	3.18 (2.46, 3.89)	0.007	0.154
GLU, mmol/L	8.20 (6.23, 12.39)	8.05 (6.13, 12.25)	8.80 (6.63, 13.07)	0.004	0.165
HBA1C, %	9.00 (7.20, 11.10)	8.90 (7.20, 11.00)	9.30 (7.40, 11.50)	0.132	0.089

BMI, body mass index; SBP, systolic blood pressure; DBP, diastolic blood pressure; HR, heart rate; WBC, white blood cell count; RBC, red blood cell count; MCV, mean red blood cell volume; LYMPH, lymphocyte count; MONO, monocyte count; NEUT, neutrophil count; EOS, eosinophil count; BASO, basophil count; HB, haemoglobin; PLT, platelet count; MPV, mean platelet volume; PDW, platelet distribution width; ALT, alanine aminotransferase; AST, aspartate aminotransferase; GGT, gamma-glutamyl transpeptidase; TBIL, total bilirubin; DBIL, direct bilirubin; IBIL, indirect bilirubin; ALB, albumin; GLO, globulin; TP, total protein; CREA, creatinine; UA, uric acid; TC, total cholesterol; TG, triglycerides; HDL-C, high-density lipoprotein cholesterol; LDL-C, low-density lipoprotein cholesterol; GLU, glucose; HBA1C, glycosylated haemoglobin.

**Figure 2:**
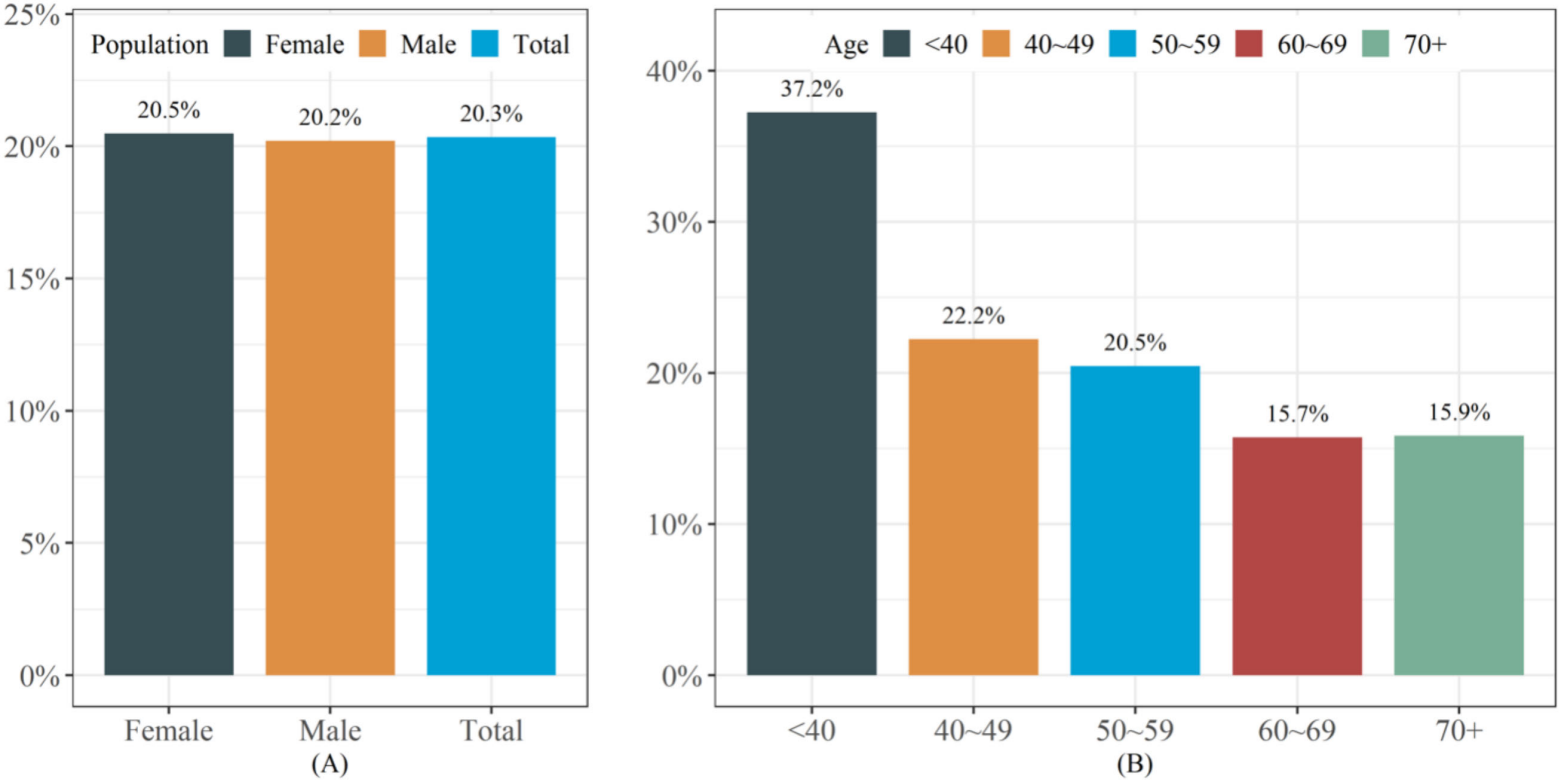
The prevalence of lean NAFLD in the study population. **(A)** The prevalence of lean NAFLD in different populations **(B)** The prevalence of lean NAFLD in different age groups.

**Figure 2** presented the prevalence rate of lean NAFLD in the study population. The overall prevalence rate of lean NAFLD is 20.3%, with the prevalence rate among females (20.5%) being slightly higher than that among males (20.2%) (**Figure 2A**). Age pattern analysis showed that the prevalence of lean NAFLD decreased with increasing age (**Figure 2B**).

### Independent risk factors

**Figure 3:**
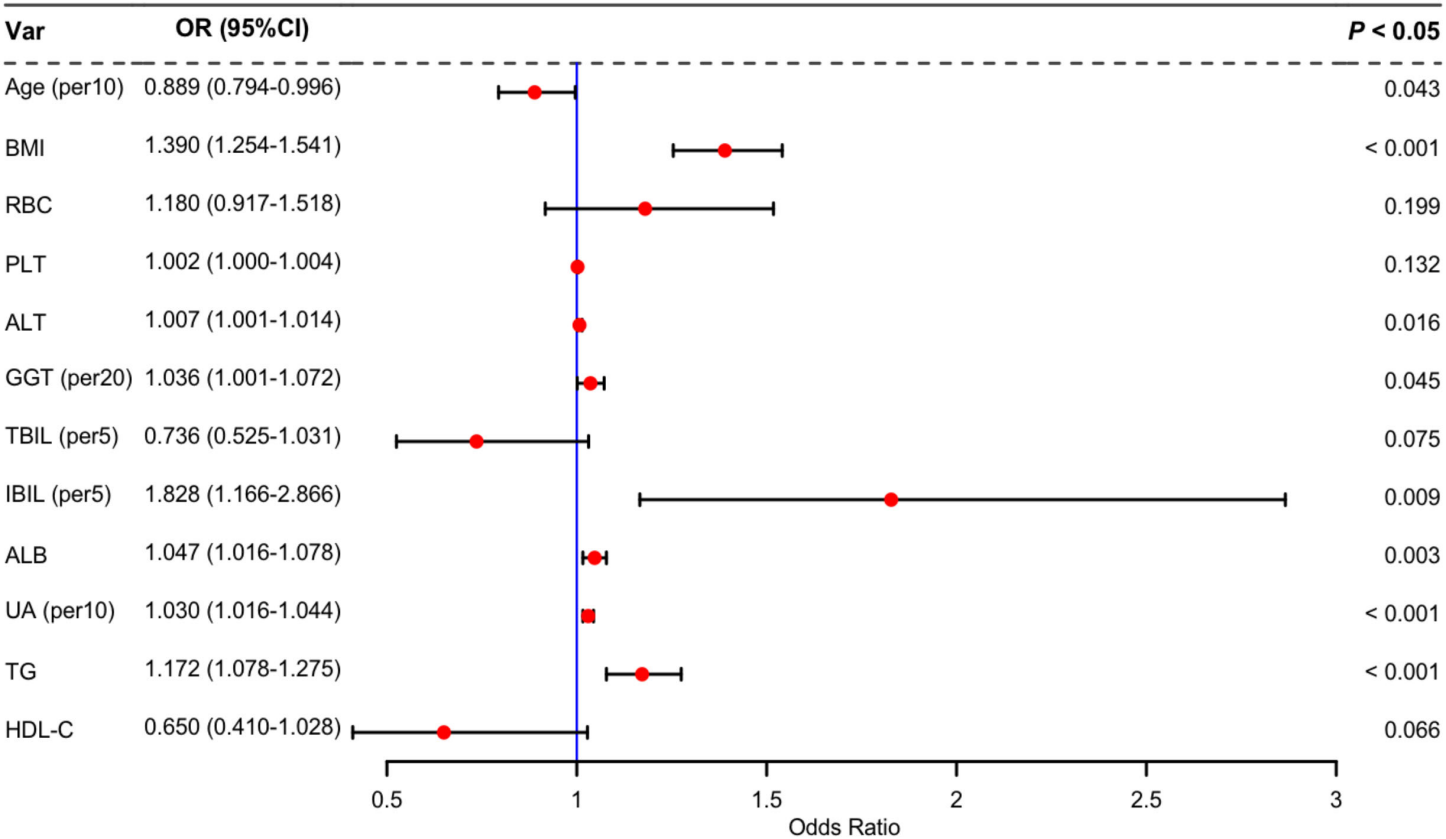
Multivariate logistic regression analysis of lean NAFLD.

Twenty potential risk factors associated with lean NAFLD were screened through univariate analysis (*p* < 0.05 and SMD > 0.1) (**Table 1**). To ensure the accuracy and reliability of the research results, the variance inflation factor (VIF) of each variable was calculated. Variables with a VIF value exceeding 10 typically exhibit severe multicollinearity (**Supplementary Table 1**). Subsequently, we employed stepwise backward logistic regression analysis with the Akaike information criterion to filter and remove multicollinear variables. Ultimately, twelve variables were included for the multivariate logistic regression analysis, and eight variables such as Age, BMI, ALT, GGT, IBIL, ALB, UA, and TG were identified as independent risk factors for lean NAFLD (*p* < 0.05). The results were presented in **Figure 3**.

### Predictor screening and construction of risk prediction models

The study population was divided into a training set and an internal validation set at a ratio of 6:4. Statistical analysis revealed no significant differences between the two groups (**Table 2**).

**Table 2:** Characteristics of participants in different sets.

	Overall	Training set	Internal validation set	*P*-value
N	1553	1089	464	
Gender (Male), %	713 (45.9)	485 (44.5)	228 (49.1)	0.107
Age, years	59.00 (50.00, 67.00)	59.00 (50.00, 66.00)	58.00 (50.00, 67.00)	0.981
BMI, kg/m^2^	22.03 (20.76, 23.05)	22.03 (20.75, 23.03)	22.06 (20.76, 23.05)	0.427
SBP, mmHg	130.00 (118.00, 144.00)	129.00 (118.00, 144.00)	131.00 (118.00, 145.25)	0.233
DBP, mmHg	77.00 (70.00, 85.00)	77.00 (70.00, 84.00)	78.00 (70.00, 85.00)	0.412
HR, n	81.00 (75.00, 92.00)	81.00 (74.00, 92.00)	82.00 (75.00, 91.00)	0.459
Smoking (Yes), %	271 (17.5)	180 (16.5)	91 (19.6)	0.164
WBC, ×10^9^/L	6.53 (5.35, 7.90)	6.58 (5.40, 8.00)	6.50 (5.25, 7.70)	0.139
RBC, ×10^12^/L	4.47 (4.11, 4.87)	4.47 (4.10, 4.88)	4.46 (4.14, 4.85)	0.558
MCV, fL	90.60 (88.00, 93.40)	90.60 (88.00, 93.40)	90.60 (88.00, 93.60)	0.866
LYMPH, ×10^9^/L	1.70 (1.30, 2.20)	1.70 (1.30, 2.20)	1.80 (1.40, 2.20)	0.613
MONO, ×10^9^/L	0.40 (0.31, 0.53)	0.40 (0.31, 0.50)	0.40 (0.32, 0.59)	0.141
NEUT, ×10^9^/L	4.00 (3.20, 5.20)	4.00 (3.20, 5.30)	4.00 (3.08, 5.00)	0.094
ESO, ×10^9^/L	0.08 (0.04, 0.14)	0.08 (0.04, 0.14)	0.09 (0.04, 0.14)	0.566
BASO, ×10^9^/L	0.03 (0.02, 0.04)	0.03 (0.02, 0.04)	0.03 (0.02, 0.04)	0.018
HB, g/L	130.00 (113.00, 143.00)	131.00 (114.00, 144.00)	129.00 (109.75, 140.00)	0.036
PLT, ×10^9^/L	213.00 (177.00, 252.00)	215.00 (177.00, 254.00)	209.00 (179.00, 250.00)	0.432
MPV, fL	10.30 (9.60, 11.00)	10.30 (9.60, 11.00)	10.30 (9.60, 11.00)	0.751
PDW, %	12.10 (10.80, 13.90)	12.10 (10.80, 13.90)	11.95 (10.70, 13.90)	0.849
ALT, IU/L	19.00 (13.00, 28.00)	19.00 (14.00, 28.00)	18.00 (13.00, 28.00)	0.763
AST, IU/L	19.00 (16.00, 25.00)	19.00 (16.00, 25.00)	19.00 (16.00, 24.25)	0.823
GGT, U/L	21.00 (15.00, 34.00)	21.00 (15.00, 34.00)	20.00 (15.00, 34.00)	0.413
TBIL, μmol/L	10.60 (8.00, 14.10)	10.70 (8.20, 14.10)	10.50 (7.79, 14.12)	0.473
DBIL, μmol/L	3.00 (2.30, 4.00)	3.00 (2.30, 4.00)	3.00 (2.20, 3.90)	0.41
IBIL, μmol/L	7.50 (5.40, 10.20)	7.51 (5.50, 10.30)	7.25 (5.20, 10.20)	0.513
ALB, g/L	41.30 (37.80, 44.70)	41.20 (37.70, 44.70)	41.35 (37.90, 44.82)	0.873
GLO, g/L	28.10 (25.40, 31.50)	28.10 (25.30, 31.50)	28.10 (25.50, 31.30)	0.755
TP, g/L	69.90 (65.00, 74.50)	69.90 (65.20, 74.60)	69.80 (64.60, 74.12)	0.625
CREA, μmol/L	60.00 (50.00, 74.00)	60.00 (50.00, 75.00)	60.00 (51.00, 73.00)	0.988
UA, μmol/L	295.00 (240.60, 357.00)	294.80 (241.00, 357.10)	295.90 (239.45, 355.62)	0.885
TC, mmol/L	4.76 (4.01, 5.66)	4.76 (4.01, 5.66)	4.78 (4.01, 5.68)	0.899
TG, mmol/L	1.30 (0.91, 1.91)	1.30 (0.92, 1.90)	1.29 (0.89, 1.93)	0.595
HDL-C, mmol/L	1.16 (0.99, 1.39)	1.17 (0.98, 1.39)	1.16 (1.02, 1.40)	0.417
LDL-C, mmol/L	3.05 (2.42, 3.66)	3.05 (2.42, 3.66)	3.04 (2.44, 3.68)	0.943
GLU, mmol/L	8.20 (6.23, 12.39)	8.19 (6.15, 12.58)	8.24 (6.39, 11.88)	0.988
HBA1C, %	9.00 (7.20, 11.10)	9.00 (7.20, 11.20)	8.80 (7.20, 10.90)	0.58

BMI, body mass index; SBP, systolic blood pressure; DBP, diastolic blood pressure; HR, heart rate; WBC, white blood cell count; RBC, red blood cell count; MCV, mean red blood cell volume; LYMPH, lymphocyte count; MONO, monocyte count; NEUT, neutrophil count; EOS, eosinophil count; BASO, basophil count; HB, haemoglobin; PLT, platelet count; MPV, mean platelet volume; PDW, platelet distribution width; ALT, alanine aminotransferase; AST, aspartate aminotransferase; GGT, gamma-glutamyl transpeptidase; TBIL, total bilirubin; DBIL, direct bilirubin; IBIL, indirect bilirubin; ALB, albumin; GLO, globulin; TP, total protein; CREA, creatinine; UA, uric acid; TC, total cholesterol; TG, triglycerides; HDL-C, high-density lipoprotein cholesterol; LDL-C, low-density lipoprotein cholesterol; GLU, glucose; HBA1C, glycosylated haemoglobin.

**Figure 4:**
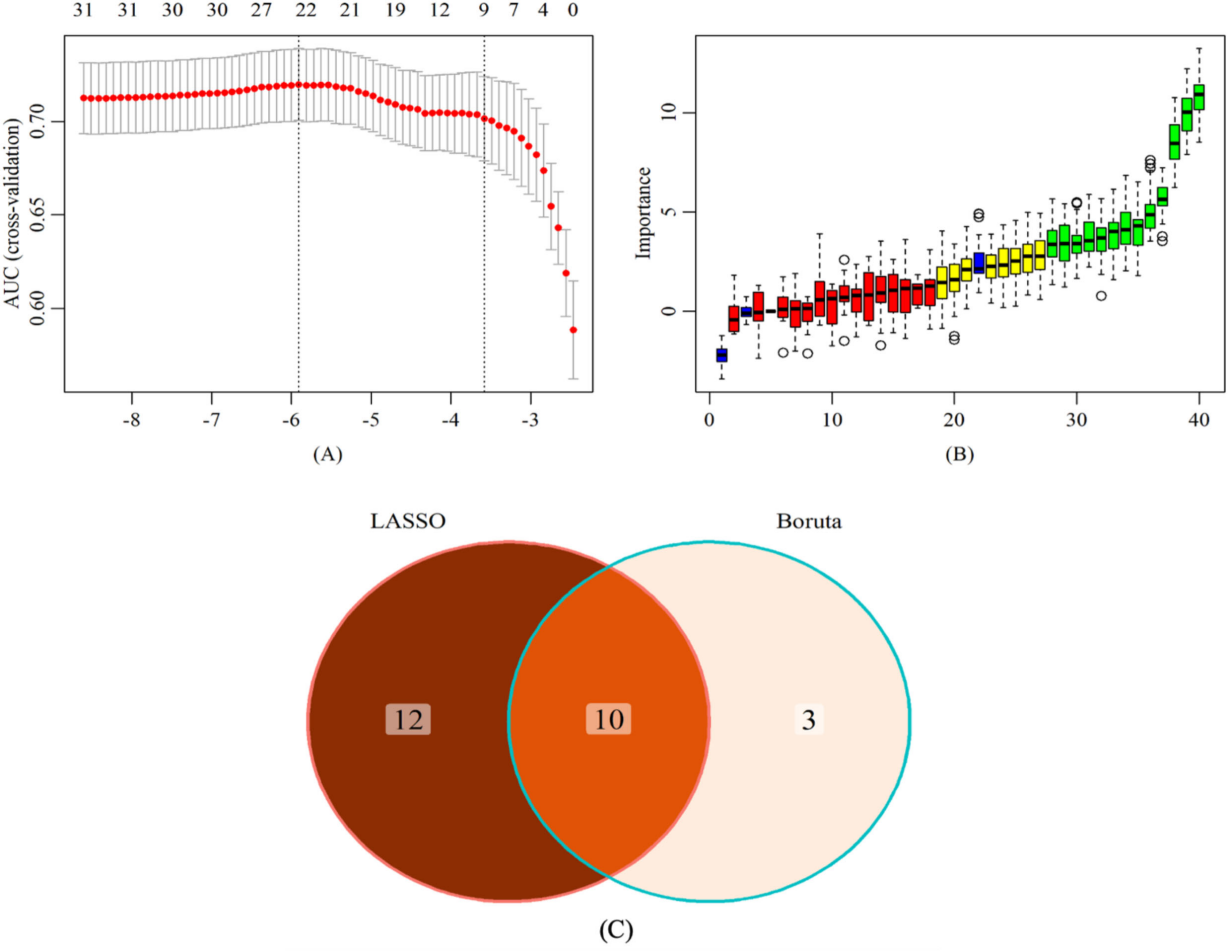
Screening of characteristic predictors. **(A)** Characteristic variables screening based on LASSO **(B)** Characteristic variables screening based on Boruta **(C)** LASSO combined Boruta. LASSO, least absolute shrinkage and selection operator.

LASSO regression is a data reduction method that reduces the complexity of the model, prevents overfitting, and selects important feature variables by formulating an optimized objective function with a penalty term (28). In this study, 22 characteristic factors were identified by using LASSO regression (**Figure 4A**).

The Boruta algorithm is a feature selection method based on random forests, aiming to identify truly significant features from a given feature set and distinguish irrelevant features (29). Thirteen key factors were identified through the Boruta algorithm (**Figure 4B**).

In the training set, through a comparative analysis of the screening results of the LASSO regression and the Boruta algorithm, we identified the common subset of feature variables selected by the two methods (**Figure 4C**). These selected variables were used as predictors to construct a risk prediction model for lean NAFLD, including BMI, LYMPH, HB, ALT, AST, GGT, IBIL, ALB, UA, and TG. In addition, the optimal model was determined among the risk prediction models constructed by six machine learning algorithms, namely LDA, LR, NB, RF, SVM, and XGboost.

### Model performance

**Figure 5:**
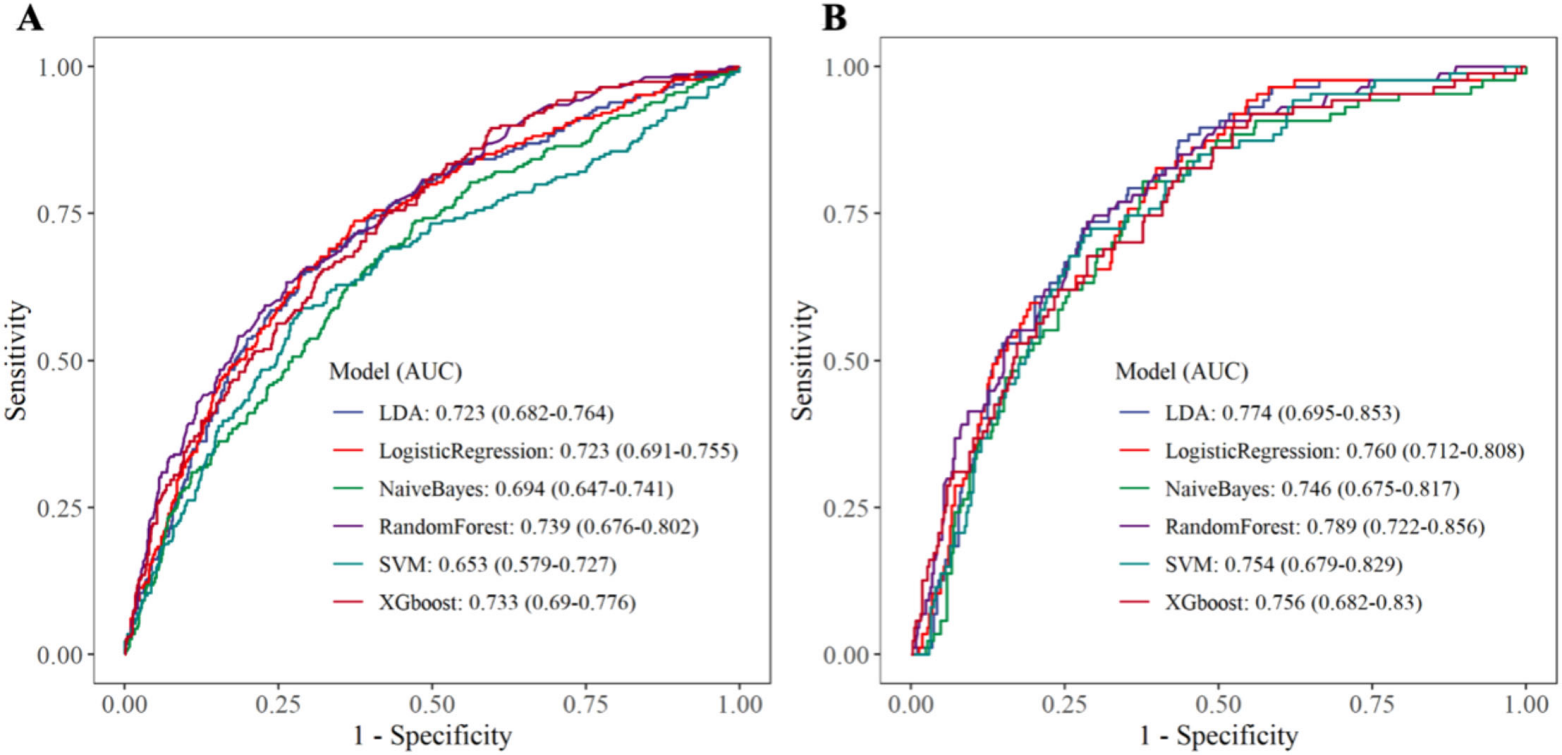
Receiver operating characteristic curve. **(A)** Training set **(B)** Internal validation set.

**Figure 6:**
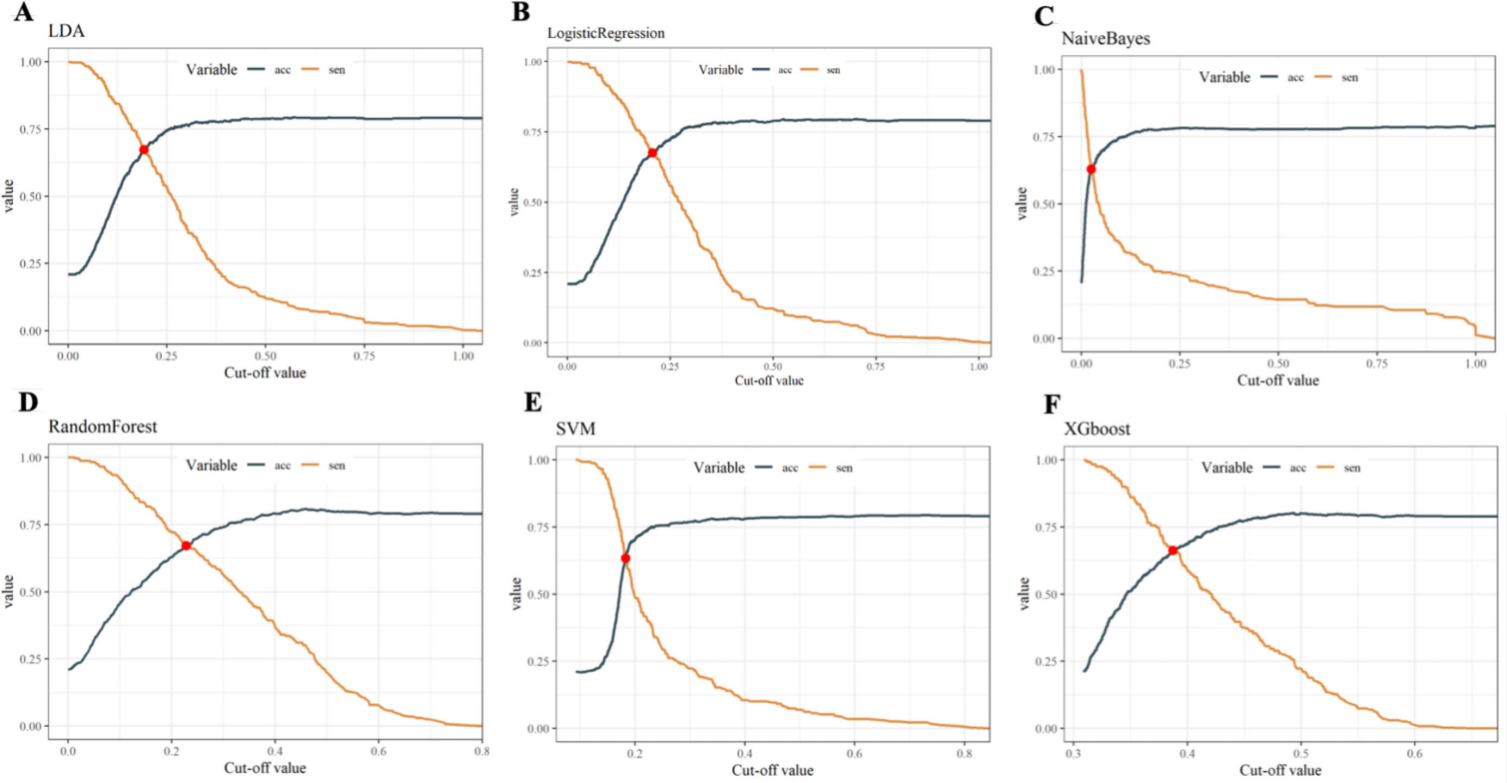
Cut-off values of six ML models in both the training set and internal validation set. **(A)** Linear discriminant analysis **(B)** Logistic regression **(C)** Naive Bayes **(D)** Random forest **(E)** Support vector machine **(F)** Extreme gradient boosting.

**Figure 7:**
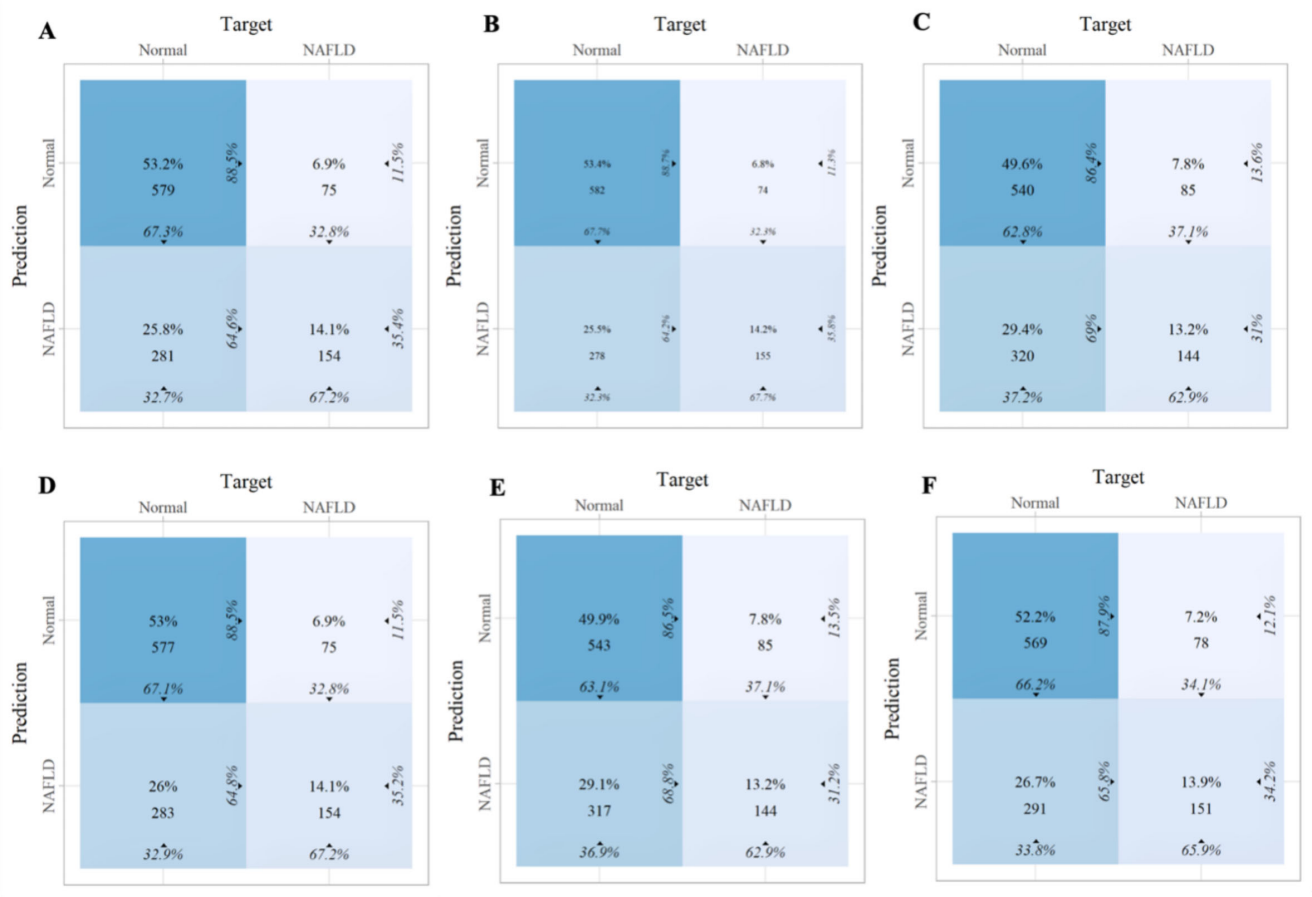
The confusion matrix of the six machine learning models in the training set. **(A)** Linear discriminant analysis **(B)** Logistic regression **(C)** Naive Bayes **(D)** Random forest **(E)** Support vector machine **(F)** Extreme gradient boosting.

**Figure 8:**
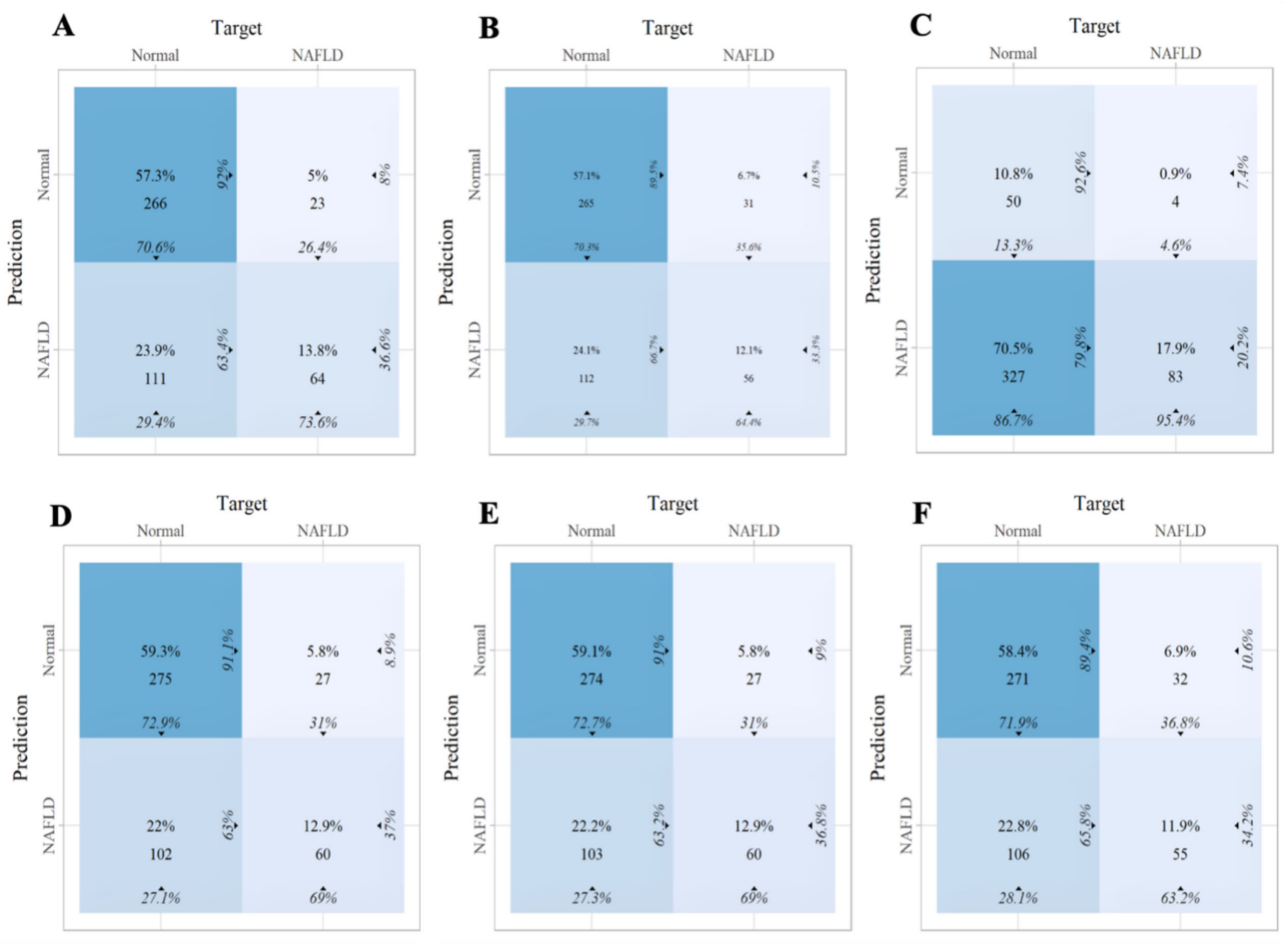
The confusion matrix of the six machine learning models in the internal validation set. **(A)** Linear discriminant analysis **(B)** Logistic regression **(C)** Naive Bayes **(D)** Random forest **(E)** Support vector machine **(F)** Extreme gradient boosting.

Within the training set, the RF model exhibited outstanding predictive performance [AUC: 0.739 (95%CI: 0.676–0.802)]. In contrast, the AUC values of the remaining five models were as follows: 0.723 (95%CI: 0.682–0.764) for LDA, 0.723 (95%CI: 0.691–0.755) for LR, 0.694 (95%CI: 0.647–0.741) for NB, 0.635 (95%CI: 0.579–0.727) for SVM, and 0.733 (95%CI:0.69–0.776) for XGboost (**Figure 5A**). In the internal validation set, the RF model also demonstrated robust clinical predictive value [AUC: 0.789 (95%CI: 0.722–0.856)] (**Figure 5B**). Furthermore, we conducted a comprehensive comparative analysis of additional clinical performance metrics, including accuracy, sensitivity, specificity, precision, recall, and F1 score, across various predictive models in both the training set and internal validation set (**Tables 3**, **4**). The table revealed that the RF model demonstrates superior performance across all evaluated parameters. Concurrently, cut-off values were respectively established for the predictive probabilities of six ML models in both the training set and internal validation set (**Figure 6**). Patients were classified as positive if the predicted probability exceeded the cut-off value; otherwise, they were categorized as negative. Consequently, confusion matrixes of the predicted probabilities and the actual values were plotted, as illustrated in the **Figures 7**, **8**.

**Table 3:** Performance parameters of six machine learning prediction models in the training set.

Model	Accuracy	Sensitivity	Specificity	Precision	Recall	F1
LR	0.677	0.677	0.677	0.358	0.677	0.468
NB	0.628	0.629	0.628	0.31	0.629	0.416
SVM	0.628	0.629	0.628	0.31	0.629	0.416
RF	0.671	0.672	0.671	0.352	0.672	0.462
XGboost	0.661	0.659	0.662	0.342	0.659	0.45
LDA	0.673	0.672	0.673	0.354	0.672	0.464

LR, logistic regression; NB, Naive Bayes; SVM, support vector machine; RF, random forest; XGboost, extreme gradient boosting; LDA, linear discriminant analysis.

**Table 4:** Performance parameters of six machine learning prediction models in the internal validation set.

Model	Accuracy	Sensitivity	Specificity	Precision	Recall	F1
LR	0.692	0.644	0.703	0.333	0.644	0.439
NB	0.287	0.954	0.133	0.202	0.954	0.334
SVM	0.72	0.69	0.727	0.368	0.69	0.48
RF	0.722	0.69	0.729	0.37	0.69	0.482
XGboost	0.703	0.632	0.719	0.342	0.632	0.444
LDA	0.703	0.632	0.719	0.342	0.632	0.444

LR, logistic regression; NB, Naive Bayes; SVM, support vector machine; RF, random forest; XGboost, extreme gradient boosting; LDA, linear discriminant analysis.

**Figure 9:**
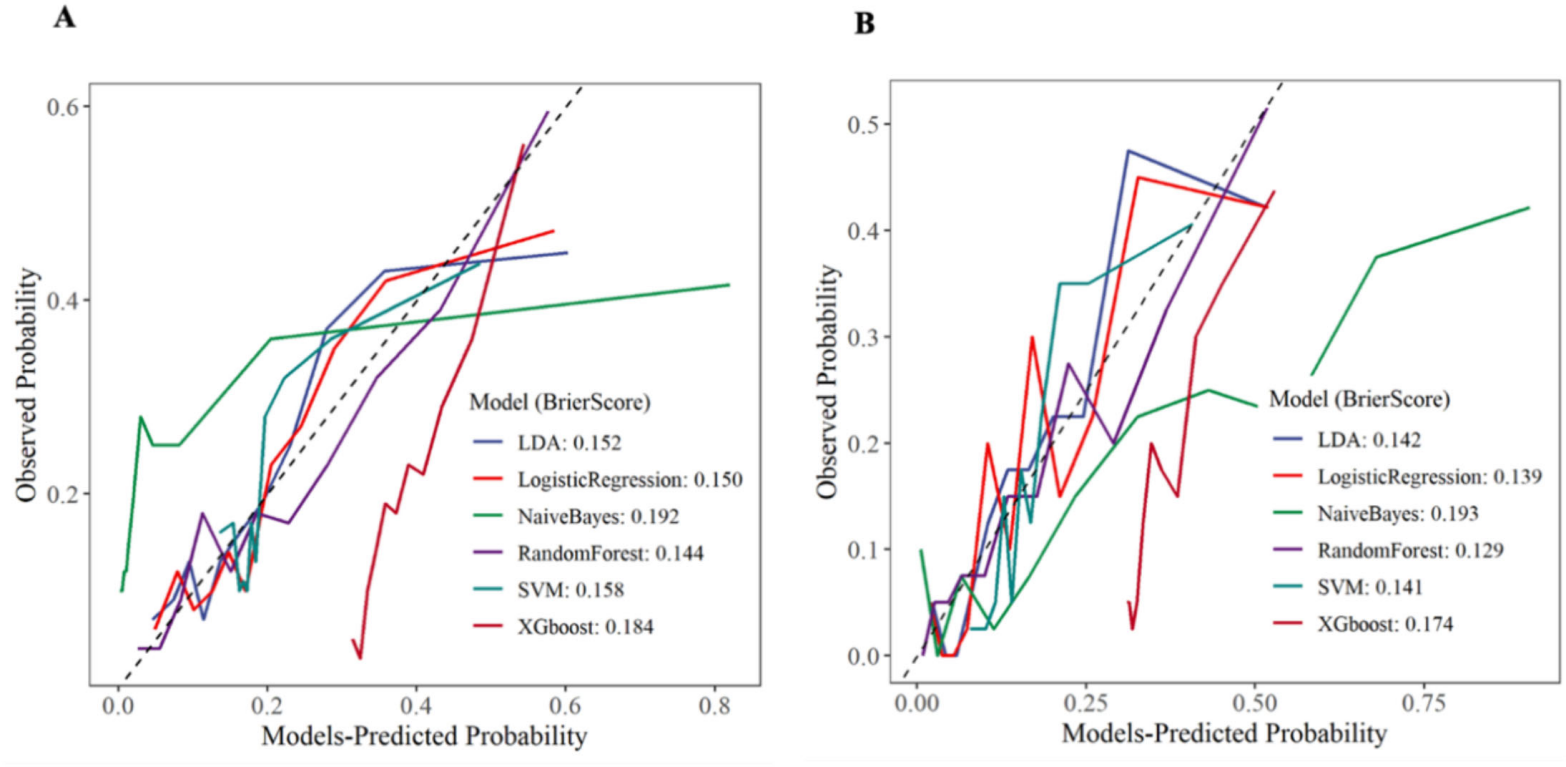
Calibration curve. **(A)** Training set **(B)** Internal validation set.

In this study, we evaluated the consistency between the model’s predicted probabilities and the actual occurrence probabilities through the analysis of calibration curves for the training set and the internal validation set. As shown in **Figure 9**, in both the training set and the internal validation set, except for the NB and XGboost models, the predicted values of the remaining models were in good agreement with the theoretical values, indicating favorable clinical calibration. Among them, the Brier score of RF model was the smallest, which reflected the high reliability of the model’s prediction.

**Figure 10:**
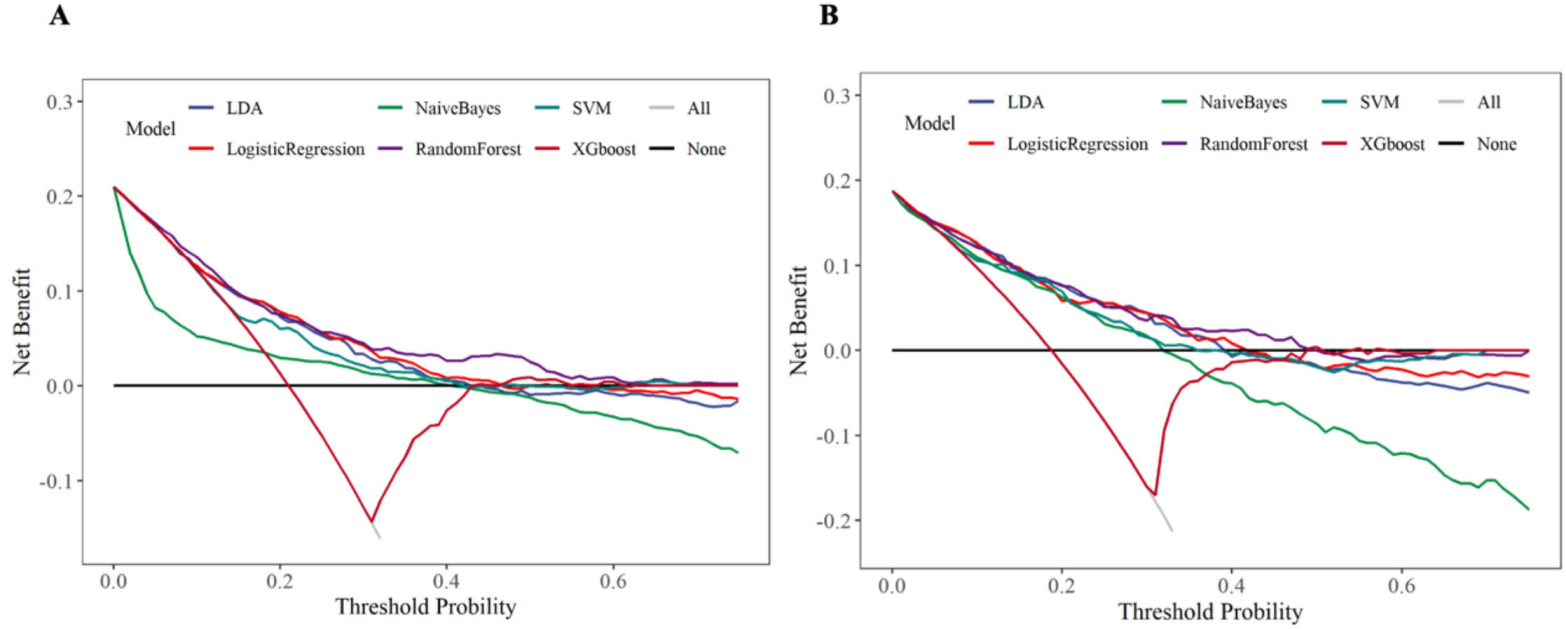
Decision curve analysis. **(A)** Training set **(B)** Internal validation set.

The DCA curve assessed the clinical decision-making value of the model under different thresholds through the net benefit. In the training set and internal validation set, except for the NB and XGboost models, the remaining models (especially the RF model) exhibited favorable clinical decision-making value. The results were presented in **Figure 10**. We further calculated the risk threshold probabilities of the RF model in the training set. The results indicated that when the threshold probability ranged between 1% and 55%, the net benefit provided by the model was significantly higher than that of the baseline strategy. In the internal validation set, the model also exhibited favorable net benefits, particularly demonstrating clinical advantages within the threshold probability range of 1% to 50%.

### SHAP-based model interpretability analysis

**Figure 11:**
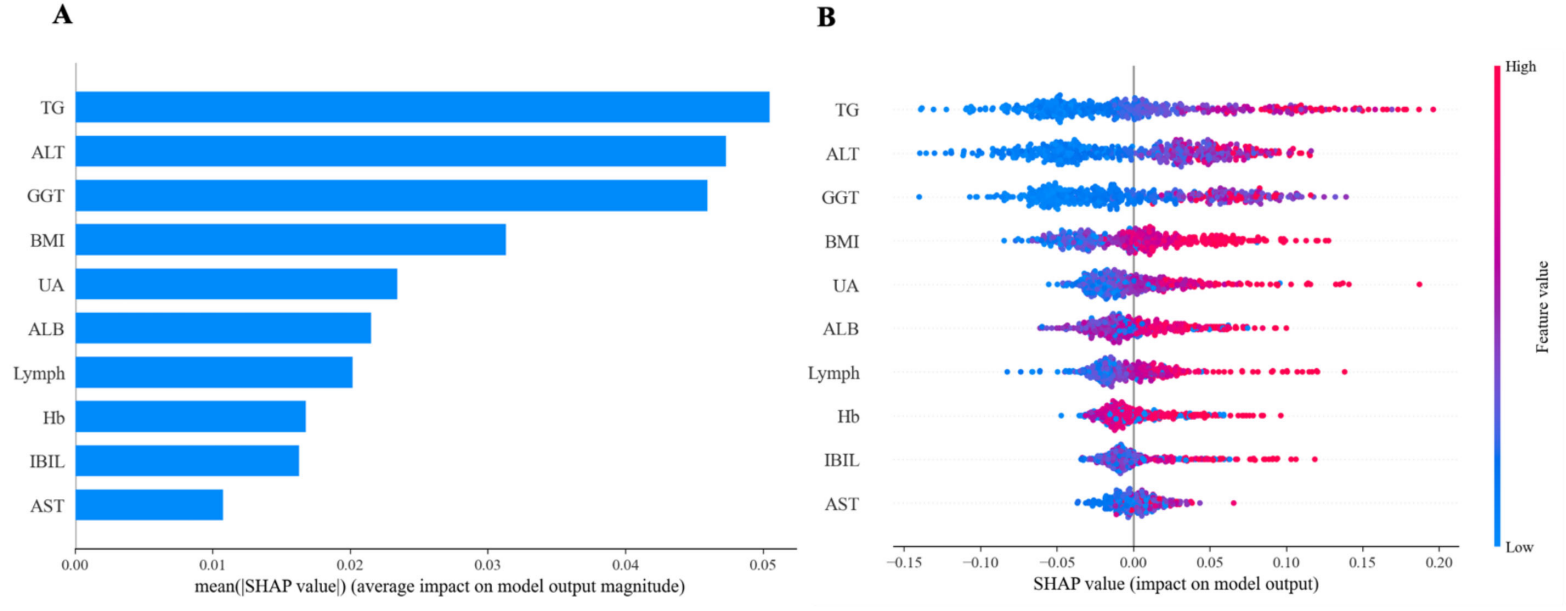
Feature importance of random forest model. **(A)** The importance ranking of the features according to the mean absolute SHAP value **(B)** The effect of features on the outcome of the model.

ML models achieve favorable performance by capturing data patterns through intricate mathematical structures. However, their complexity makes it difficult to interpret their internal decision - making processes, and they are commonly regarded as “black-box” models. SHAP is a tool designed for interpreting machine learning models. It explains the prediction results of a model by assigning a “contribution value” (Shapley value) to each feature, thereby rendering the decision-making process of black-box models transparent and controllable (30). Based on the above results, the RF model emerged as the optimal predictive model, demonstrating stable performance in both the training set and the internal validation set. Therefore, we further employed interpretable tools to analyze the contributions of characteristic predictors to the RF model. **Figure 11A** illustrated the contribution of characteristic predictors to the prediction model in the RF model, where TG, ALT, GGT, BMI, and UA were the top five variables in terms of importance. From **Figure 11B**, we could observe that the larger the TG, ALT, GGT, BMI, and UA, the greater the SHAP value and the higher the risk of disease.

## Discussion

A total of 1,553 patients with T2DM were included in this study. Ultimately, 316 patients (20.3%) were diagnosed with lean NAFLD. The prevalence rate was lower than the 33.18% reported by Zhang X et al. (31), which might be associated with sample size and the region. Multivariate logistic regression analysis revealed that eight variables including Age, BMI, ALT, GGT, IBIL, ALB, UA, and TG, were independent risk factors for lean NAFLD in patients with T2DM (*p* < 0.05), which was generally consistent with the findings of previous studies (32–35). We employed a dual methodology of Boruta’s algorithm and LASSO regression to identify ten predictors for accurate feature selection and model stability, namely BMI, LYMPH, HB, ALT, AST, GGT, IBIL, ALB, UA, and TG. These variables have all been shown to be related to lean NAFLD in previous studies (13, 36, 37). Meanwhile, we established and validated the clinical performance of six ML models. The results showed that the RF model demonstrated the highest clinical predictive value in the training set [AUC: 0.739 (95%CI: 0.676–0.802)] and performed exceptionally well in terms of accuracy, sensitivity, specificity, precision, recall, and F1 score. This was consistent with the results of the study by M et al. on the evaluation of diabetes using ML techniques (38). The SHAP explanation of the RF model showed that TG, ALT, GGT, BMI, and UA were the top five most contributive variables in the predictive model.

Most patients with T2DM are the potential population at risk of lean NAFLD. Patients with T2DM are particularly prone to non-alcoholic steatohepatitis (NASH) and face a higher risk of progressing to liver cirrhosis and hepatocellular carcinoma (39, 40). Research indicated that lipid metabolism in the liver has already been disrupted during the pre-diabetic stage (41). Insulin resistance (IR) plays a crucial role in the occurrence and development of T2DM complicated with lean NAFLD. IR in adipose tissue increases lipolysis and the release of free fatty acids (FFA) and glycerol, leading to the accumulation of triglycerides in the liver. Subsequently, chronic inflammation, up-regulation of hepatotoxic cytokines, oxidative stress, and alterations in the gut microbiota, which damage the liver and cause it to develop into NASH (42–44). Among them, the dysregulation of the spleen-liver immune axis serves as a crucial driving force for the progression of lean NAFLD. Specifically, FFA and pro-inflammatory factors (such as TNF-α and IL-6) enter the spleen via the portal vein circulation, leading to the activation of splenic natural killer T (NKT) cells and the accumulation of myeloid - derived suppressor cells (MDSCs). These abnormally activated immune cells and inflammatory mediators migrate to the liver through the portal vein. On one hand, they exacerbate IR in hepatocytes by inhibiting the insulin signaling pathway. On the other hand, they activate hepatic Kupffer cells, thereby inducing chronic inflammatory responses (45, 46).

Therefore, the identification and screening of lean NAFLD should be incorporated into the routine treatment of patients with T2DM. Due to the lack of reliable methods for detecting steatosis, lean NAFLD is mostly incidentally detected during imaging examinations. However, the large-scale promotion of imaging examinations will inevitably lead to the waste of medical resources and an increase in time costs. Serum indices and imaging techniques are employed for the general screening of diabetic patients to detect advanced fibrosis. However, Qadri S et al. found that serum indices lack high specificity and imaging techniques yield a high rate of false positives (47). In this study, we developed a risk prediction model for lean NAFLD in patients with T2DM based on interpretable ML algorithms. The aim was to identify high-risk individuals at an early stage, thereby reducing the occurrence of adverse events and optimizing medical resources.

This study has certain limitations. Firstly, the sample size of this study was relatively small and confined to a specific populations and regions, which may limit the generalizability of the research findings. Secondly, the collection of clinical data was incomplete, potentially leaving out some latent predictive factors. Finally, the risk prediction model had only been validated using the internal dataset, without external dataset validation or temporal validation. Therefore, in future research, it is advisable to integrate multi-center data and utilize more advanced machine learning techniques to enhance the performance of the model.

## Conclusion

This study has effectively developed a risk prediction model for lean NAFLD in patients with T2DM, which holds significant clinical implications for reducing and preventing adverse events. Among them, the performance of the RF model outperforms that of other ML algorithms.

## Data Availability

The original contributions presented in the study are included in the article/**Supplementary Material**. Further inquiries can be directed to the corresponding authors.

## Glossary

ALB: albumin
ALT: alanine aminotransferase
AST: aspartate aminotransferase
AUC: area under the receiver operating characteristic curve
BMI: body mass index
DCA: decision curve analysis
FFA: free fatty acids
GGT: gamma-glutamyl transpeptidase
HB: hemoglobin
IBIL: indirect bilirubin
IR: Insulin resistance
LASSO: Least Absolute Shrinkage and Selection Operator
LDA: linear discriminant analysis
LR: logistic regression
LYMPH: lymphocyte count
ML: machine learning
NAFLD: Non-alcoholic fatty liver disease
NASH: non-alcoholic steatohepatitis
NB: Naive Bayes
RF: random forest
SHAP: Shapley Additive exPlanations
SVM: support vector machine
TBIL: total bilirubin
T2DM: type 2 diabetes mellitus
TG: triglycerides
UA: uric acid
VIF: variance inflation factor
XGboost: extreme gradient boosting
