# Supplementary material for: Predicting the risk of lean non-alcoholic fatty liver disease based on interpretable machine models in a Chinese T2DM population
Source: Front Endocrinol (Lausanne). 2025 Jul 11;16:1626203. doi: 10.3389/fendo.2025.1626203 (PMC12289481; doi:10.3389/fendo.2025.1626203)
Supplement: Supplementary file 1 [file DataSheet1.pdf]

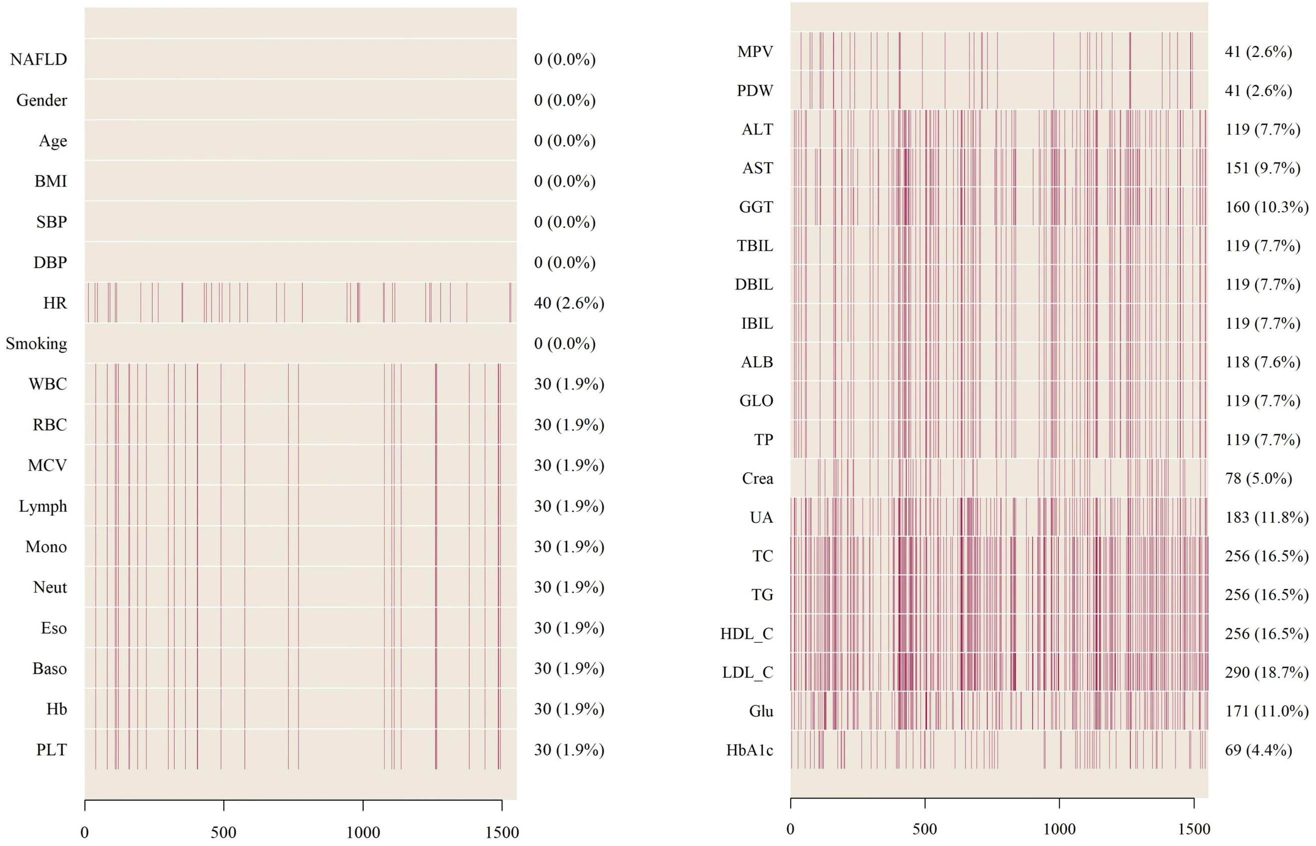

Figure S1 Missing value of total data

Table S1 Variance inflation factors for candidate independent risk factors

N	VIF
Age	1.199196171
BMI	1.042008053
RBC	1.353546888
PLT	1.079551219
ALT	1.350857854
GGT	1.523922027
TBIL	4.296888235
IBIL	3.831845116
ALB	1.248011883
UA	1.090195009
TG	1.156119174
HDL-C	1.100554903

VIF variance inflation factor, BMI body mass index, RBC red blood cell count, PLT platelet count, ALT alanine aminotransferase, GGT gamma-glutamyl transpeptidase, TBIL total bilirubin, IBIL indirect bilirubin, ALB albumin, UA uric acid, G triglycerides, HDL-C high-density lipoprotein cholesterol
